# The Impact of Breathing Hypoxic Gas and Oxygen on Pulmonary Hemodynamics in Patients With Pulmonary Hypertension

**DOI:** 10.3389/fmed.2022.791423

**Published:** 2022-02-11

**Authors:** Arcangelo F. Carta, Mona Lichtblau, Charlotte Berlier, Stéphanie Saxer, Simon R. Schneider, Esther I. Schwarz, Michael Furian, Konrad E. Bloch, Silvia Ulrich

**Affiliations:** Department of Pulmonology, University Hospital Zurich, University of Zurich, Zurich, Switzerland

**Keywords:** pulmonary arterial hypertension (PAH), chronic thromboembolic pulmonary arterial hypertension (CTEPH), right heart catheterization, oxygen, hypoxia, right to left shunting

## Abstract

**Background:**

Pure oxygen breathing (hyperoxia) may improve hemodynamics in patients with pulmonary hypertension (PH) and allows to calculate right-to-left shunt fraction (Qs/Qt), whereas breathing normobaric hypoxia may accelerate hypoxic pulmonary vasoconstriction (HPV). This study investigates how hyperoxia and hypoxia affect mean pulmonary artery pressure (mPAP) and pulmonary vascular resistance (PVR) in patients with PH and whether Qs/Qt influences the changes of mPAP and PVR.

**Study Design and Methods:**

Adults with pulmonary arterial or chronic thromboembolic PH (PAH/CTEPH) underwent repetitive hemodynamic and blood gas measurements during right heart catheterization (RHC) under normoxia [fractions of inspiratory oxygen (FiO_2_) 0.21], hypoxia (FiO_2_ 0.15), and hyperoxia (FiO_2_ 1.0) for at least 10 min.

**Results:**

We included 149 patients (79/70 PAH/CTEPH, 59% women, mean ± SD 60 ± 17 years). Multivariable regressions (mean change, *CI*) showed that hypoxia did not affect mPAP and cardiac index, but increased PVR [0.4 (0.1–0.7) WU, *p* = 0.021] due to decreased pulmonary artery wedge pressure [−0.54 (−0.92 to −0.162), *p* = 0.005]. Hyperoxia significantly decreased mPAP [−4.4 (−5.5 to −3.3) mmHg, *p* < 0.001] and PVR [−0.4 (−0.7 to −0.1) WU, *p* = 0.006] compared with normoxia. The Qs/Qt (14 ± 6%) was >10 in 75% of subjects but changes of mPAP and PVR under hyperoxia and hypoxia were independent of Qs/Qt.

**Conclusion:**

Acute exposure to hypoxia did not relevantly alter pulmonary hemodynamics indicating a blunted HPV-response in PH. In contrast, hyperoxia remarkably reduced mPAP and PVR, indicating a preserved vasodilator response to oxygen and possibly supporting the oxygen therapy in patients with PH. A high proportion of patients with PH showed increased Qs/Qt, which, however, was not associated with changes in pulmonary hemodynamics in response to changes in FiO_2_.

## Background

Due to the increase of global mobility and advances in infrastructure, every year millions of people expose themselves to hypobaric hypoxic environments, such as high altitude or commercial air travel. In healthy individuals, a low inspired and alveolar oxygen concentration causes hypoxic pulmonary vasoconstriction (HPV), which in turn increases pulmonary vascular resistance (PVR) and pulmonary arterial pressure (PAP) (1). When exposed to a hypoxic environment, HPV could be detrimental in patients with pre-existing precapillary pulmonary hypertension (PH) due to pulmonary vascular disease (2–4), including pulmonary arterial and chronic thromboembolic PH (PAH and CTEPH). To date, only few studies have measured PAP and PVR in precapillary PH under hypoxia during the right heart catheterization (RHC) (5, 6).

Contrarily, acute exposure to high fractions of inspiratory oxygen (FiO_2_) may reduce PAP and PVR in patients with PH through pulmonary vasodilation, though there are only few studies with hemodynamic data obtained during RHC (6–8). The exact mechanism of pulmonary vasodilation in response to oxygen in precapillary PH remains under investigation. Some evidence suggests that the predominantly alveolar endothelial oxygen sensing and, to a lesser degree, direct oxyhemoglobin sensing in mixed venous blood are involved, but data on the influence of mixed venous oxygen partial pressures on the hemodynamic response to oxygen in patients with PH are lacking (7). Pulmonary vasodilation by oxygen supplementation might provide a physiological basis for supporting long-term oxygen therapy in PH, which was shown to improve exercise capacity and symptom relief in domiciliary and nocturnal settings (9, 10).

Breathing pure oxygen (hyperoxia, FiO_2_ = 1.0) additionally allows calculating the fraction of right-to-left shunt (Qs/Qt) (11). We hypothesized that the size of Qs/Qt may influence PAP and PVR and their response to changes in FiO_2_ in patients with precapillary PH. Right-to-left shunts are physiological or anatomical short circuits, which occur when blood flows from the right to the left side of the heart without passing through the pulmonary capillaries. Physiological shunts occur when non-ventilated alveoli are perfused (12). Anatomical right-to-left shunts are most frequently located in the heart, with the patent foramen ovale (PFO) constituting the most common pathology with an estimated prevalence of 17–30% in the general population and similarly in patients with PH (13–18), or are located in the lungs as intrapulmonary arteriovenous anastomoses (IPAVA) without direct contact to the alveolar system (19). An increased Qs/Qt might be associated with distinct exaggerated hemodynamic changes under hypoxia (3) or could reflect a potential escape valve mechanism on increased PAP.

In this study, we aim to investigate the effect of hypoxia (FiO_2_ 0.15) and hyperoxia (FiO_2_ 1.0) on pulmonary hemodynamics (mPAP, PVR) in a large cohort of patients with PH assessed by RHC as well as the influence of Qs/Qt on hemodynamic parameters, which might provide insight on physiological mechanisms supporting the use of therapeutic oxygen in this patient collective.

## Methods

### Study Design and Participants

This is a retrospective study analyzing data obtained in patients with precapillary PH due to pulmonary vascular disease (PAH/CTEPH) during clinically indicated RHC at the University Hospital Zurich, 490 m altitude. This study was conducted in concordance with the amended Helsinki declaration, approved by the cantonal ethics committee Zurich (No. 2020-01541) and all patients provided written informed consent.

We included men and women, aged ≥18 years with mean PAP (mPAP) > 20 mmHg, pulmonary artery wedge pressure (PAWP) ≤ 15 mmHg) and revealing a PVR > 2.0 WU (20–22) referred to our clinic between March 2016 and June 2020 for RHC and who had undergone measurements of hemodynamic parameters while breathing ambient air (normoxia FiO_2_ 0.21), hypoxia (FiO_2_ 0.15), and hyperoxia (FiO_2_ 1.0) and who were classified as PAH or CTEPH according to a thorough investigation, including pulmonary function tests, CT-pulmonary angiography, ventilation-perfusion scan, serology, and other measures as required. We excluded severely hypoxemic patients (PaO_2_ <7.3 kPa on ambient air) who could not undergo hypoxia testing (21), patients after lung transplantation, and patients with other PH-classifications (2).

### Measurements

Supine resting RHC was performed *via* jugular venous access with a balloon-tipped, triple-lumen, fluid-filled 7.5 French Swan-Ganz catheter (Baxter/Edwards, Deerfield, IL, USA) as described (6, 23). Transducers were placed at the mid-thoracic level and zeroed to atmospheric pressure (24, 25). Systolic, mean, and diastolic PAP, right atrial pressure (RAP), and PAWP were averaged over several respiratory cycles (Dräger, Liebefeld, Switzerland) (23, 25, 26). CO was measured by thermodilution (Baxter/Edwards, Deerfield, IL, USA) and indexed for body surface area (BSA) to obtain cardiac index (CI). Blood samples were drawn from radial artery puncture and pulmonary artery catheter tips were analyzed directly (RapidPoint 500, Siemens, Zurich, Switzerland) to obtain arterial partial pressures of oxygen and carbon dioxide (PaO_2_, PaCO_2_), arterial saturation of oxygen (SaO_2_), mixed venous partial pressure (PmvO_2_) and saturation of oxygen (SmvO_2_), hemoglobin (Hb), and lactate.

Patients were asked to rest supine for at least 10 min and baseline hemodynamic parameters and blood gas samples were obtained under normoxia (FiO_2_ 0.21). Patients then received hypoxia (FiO_2_ 0.15) over an airtight mouthpiece (AltiTrainer, SMTEC, Nyon, Switzerland) for at least 10 min, after which hemodynamics and blood gas samples were obtained a second time while still breathing hypoxia. Following a washout phase of at least 10 min, patients received pure oxygen (hyperoxia, FiO_2_ 1) for 10 min administered *via* a non-rebreathing valve from a reservoir bag (AmbuSPUR-II, Synmedic, Zurich, Switzerland), after which hemodynamics and blood samples were obtained for the third time while still breathing hyperoxia and shunt fraction was calculated with the following formula (11):


$$\begin{array}{l} \frac{Qs}{Qt}=\frac{\left(\left(0.0031\times \left({FiO}_{2}\times \left(P_{B}-P_{H2O}-{PaCO}_{2}/R\right)\right)\right)+\left({ScapO}_{2}\times Hb\times 1.34\right)\right)-\left(\left(1.34\times Hb\times SaO_{2}\right)+\left(0.0031\times PaO_{2}\right)\right)}{\left(\left(0.0031\times \left({FiO}_{2}\times \left(P_{B}-P_{H2O}-{PaCO}_{2}/R\right)\right)\right)+\left({ScapO}_{2}\times Hb\times 1.34\right)\right)\;-\;\left(\left(1.34\times Hb\times SmvO_{2}\right)\;+\;\left(0.0031\times PvO_{2}\right)\right)} \end{array}$$

The standard barometric atmospheric pressure at 490 m (P_B_) was assumed to be 717.7 mmHg, the partial pressure of water at body temperature (P_H2O_) assumed to be 47.1 mmHg at 37°C, the respiratory quotient (R) was assumed to be 0.8, and pulmonary capillary oxygen saturation (ScapO_2_) was assumed to be 100% under FiO_2_ 1.0. The factor 0.0031 is the solubility coefficient of oxygen in the blood and the factor 1.34 is the ratio of oxygen in milliliters per gram of Hb.

### Outcomes

The main outcomes consisted of the acute change of mPAP and PVR under hypoxia (FiO_2_ 0.15) and hyperoxia (FiO_2_ 1.0). Further outcomes included the size of Qs/Qt and the influence of Qs/Qt, PaO_2_, and PmvO_2_ on hemodynamic parameters (mPAP, PVR, CI, and PAWP) measured under normoxia, hypoxia, and hyperoxia, and subgroup-analysis separately for PAH and CTEPH.

The statistical analysis included all available measurements under normoxia, hypoxia, and hyperoxia. Single missing values were not replaced. All statistical analyses were performed using R Studio (version 1.2.1578, R Studio Inc., San Francisco, CA, USA). Analyses of hemodynamic measures (mPAP, PVR, PVR Index, CI, and PAWP) were performed in univariable (FiO_2_ = normoxia vs. hypoxia vs. hyperoxia) and multivariable mixed linear regression models, with Qs/Qt, PH group (PAH or CTEPH), age, sex, carbon monoxide transfer coefficient (K_CO_), PaO_2_, and PmvO_2_ as independent variables, and grouped by subject. Coefficients are expressed as mean change and 95% *CI*. We tested regression model fitting with QQ-plots and fitted residual and random intercept plots. Multi-collinearity was excluded with variance inflation factor testing. Continuous data are reported as mean ± SD and categorical data as number (percentage). A two-sided *p*-value of < 0.05 or a 95% *CI* not crossing 0 was considered statistically significant.

## Results

### Study Population

**Figure 1:**
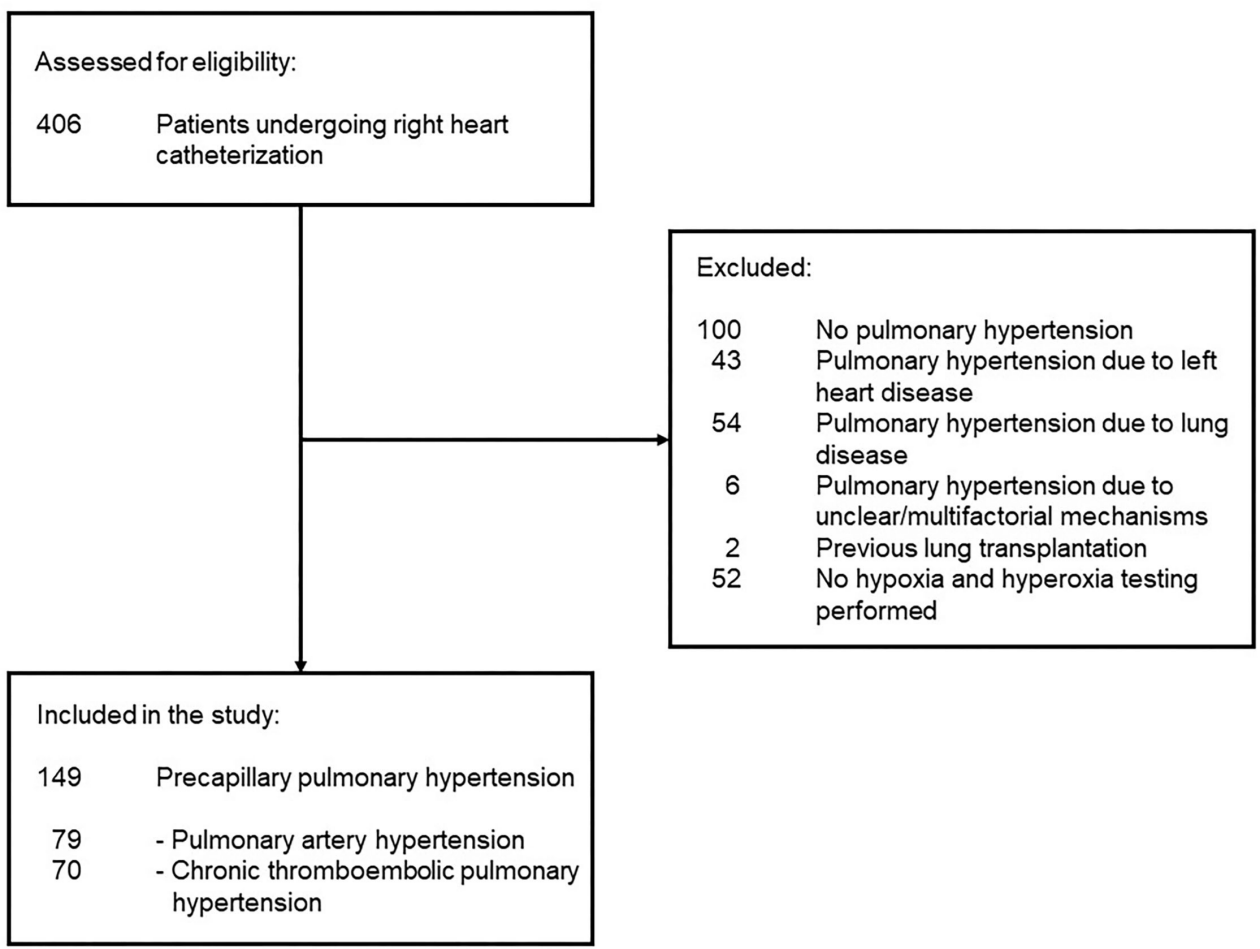
Patient flow is shown. No pulmonary hypertension classified patients with a mean pulmonary artery pressure <25 mmHg and a pulmonary vascular resistance <2 WU.

We included 149 patients with PH, 79 PAH, and 70 CTEPH (Figure 1). Patients with PAH were predominantly women (i.e., 70% women), whereas sex distribution was balanced in patients with CTEPH (47% women) (Table 1; Supplementary Table 1). The vast majority of patients were incident without any therapy (90% PAH and 87% CTEPH), a minority of patients with CTEPH had RHC 6–12 months after pulmonary endarterectomy and some patients with PAH were medically pretreated. Pulmonary function tests from the patient records revealed mildly decreased forced expiration in the first second of expiration (FEV_1_) and forced vital capacity (FVC), as well as mildly decreased diffusing capacity of lung carbon monoxide (DLCO) and K_CO_ values.

**Table 1:** Patient baseline characteristics.

Number	149
Female, *n* (%)	88 (59)
Age, years	60 ± 17
Height, cm	169 ± 10
Weight, kg	77 ± 17
BMI, kg/m^2^	26.7 ± 5.2
BSA, m^2^	1.9 ± 0.2
Pulmonary hypertension classification (WHO)	
1. Pulmonary arterial hypertension	79 (53)
1.1. Idiopathic	31 (21)
1.2. Hereditable	1 (1)
1.4.1. Connective tissue disease	35 (23)
1.4.2. HIV Infection	3 (2)
1.4.3. Portal hypertension	3 (2)
1.4.4. Congenital heart disease	5 (3)
1.4.5. Schistosomiasis	1 (1)
4.1. Chronic thromboembolic pulmonary hypertension	70 (47)
Incident patients newly diagnosed not yet on therapy: PAH/CTEPH	71 (90)/61 (87)
Pulmonary function tests, % predicted	
FEV_1_	84 ± 19
FVC	86 ± 18
VC	95 ± 20
TLC	95 ± 23
DLCO	62 ± 20
Kco	72 ± 20

*Data are displayed as mean ± SD or as number (n) with percentage (%)*.

*CTEPH, chronic thromboembolic pulmonary hypertension; DLCO, diffusion capacity of carbon monoxide; FEV_1_, forced expiration in the first second of expiration; FVC, forced vital capacity; K_CO_, carbon monoxide transfer coefficient; PAH, pulmonary arterial hypertension; TLC, total lung capacity; VC, vital capacity; WHO, World Health Organization; BMI, body mass index; BSA, body surface area*.

### Resting Hemodynamics Under Different FiO_2_

**Figure 2:**
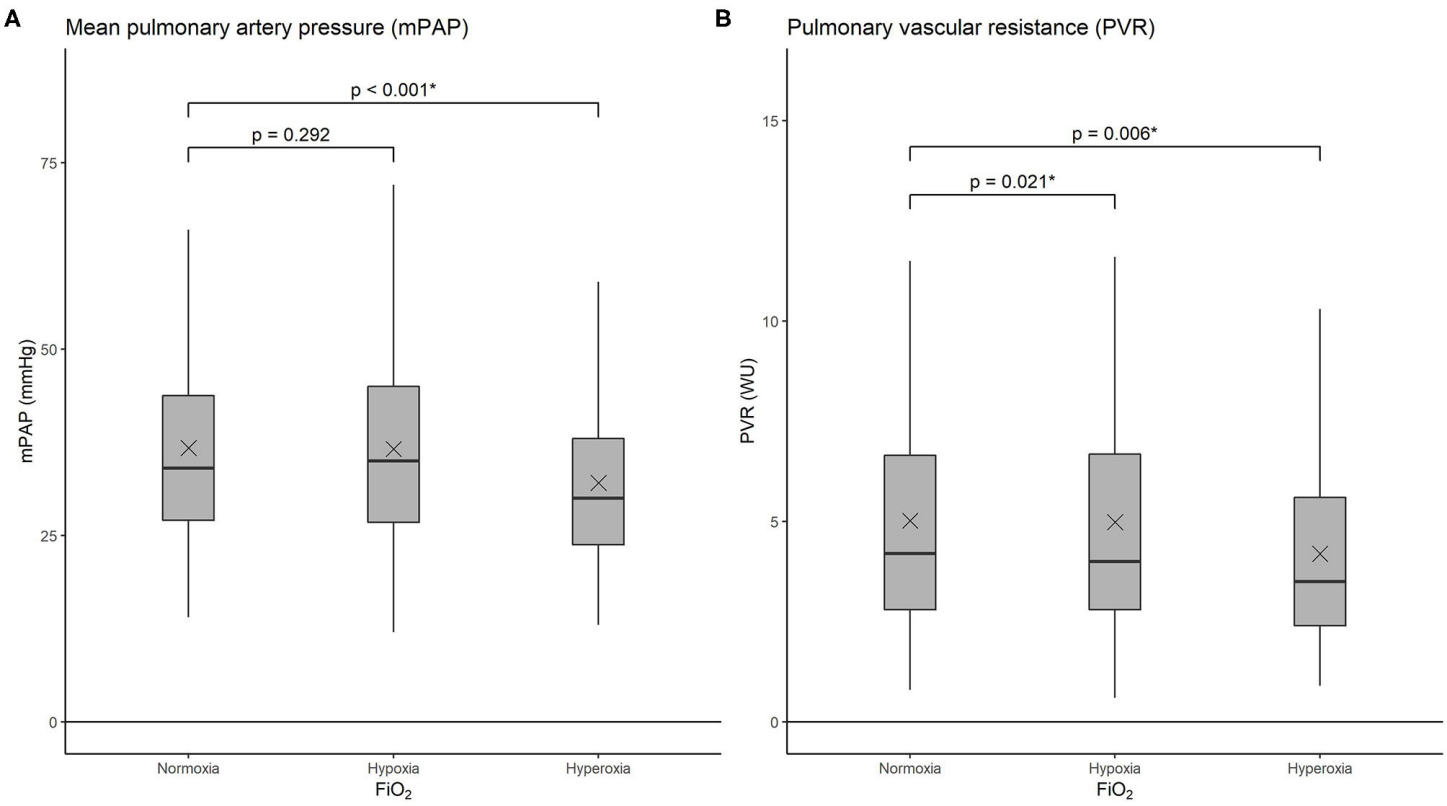
Boxplots showing mean pulmonary artery pressure (mPAP) **(A)** and pulmonary vascular resistance (PVR) **(B)** under normoxia (FiO_2_ 0.21), hypoxia (FiO_2_ 0.15), and hyperoxia (FiO_2_ 1.0). The horizontal lines represent the median values, upper and lower box edges represent the 25th and 75th percentiles, and “x” indicates the mean. FiO_2_: fraction of inspired oxygen.

Under normoxia, patients with PH showed an increased mPAP and PVR and mild hypoxemia according to inclusion criteria (Table 2 and Figure 2). Under hypoxia, a univariable mixed linear regression analysis adjusted for FiO_2_ showed that the mPAP did not change significantly compared with normoxia, while the PVR increased by 0.35 WU and the PAWP decreased by 0.5 mmHg. Arterial and mixed venous oxygen and carbon dioxide decreased.

**Table 2:** Resting hemodynamics and blood gases under normoxic, hypoxic, and hyperoxic air breathing (normoxia, hypoxia, and hyperoxia).

**Resting hemodynamics during RHC**	**Normoxia (FiO_**2**_ 0.21)**	**Hypoxia (FiO_**2**_ = 0.15)**	**Hyperoxia (FiO_**2**_ = 1.0)**
Oxygen saturation (%)	94 ± 4	89 ± 6	99 ± 2
Heart rate (min^−1^)	73 ± 12	76 ± 14	68 ± 13
Blood pressure, systolic (mmHg)	130 ± 16	132 ± 19	133 ± 19
Blood pressure, mean (mmHg)	97 ± 13	99 ± 14	100 ± 14
Blood pressure, diastolic (mmHg)	76 ± 12	78 ± 12	78 ± 15
Pulmonary artery pressure, systolic (mmHg)	54 ± 19	56 ± 21	48 ± 18
Pulmonary artery pressure, mean (mmHg)	36 ± 12	37 ± 13	32 ± 11
Pulmonary artery pressure, diastolic (mmHg)	24 ± 9	24 ± 10	21 ± 8
Pulmonary artery wedge pressure (mmHg)	12 ± 3	11 ± 4	11 ± 4
Right atrial pressure (mmHg)	8 ± 4	7 ± 4	7 ± 4
Cardiac output (l/min)	5.4 ± 1.4	5.4 ± 1.5	5.1 ± 1.5
Cardiac index (l/min/m^2^)	2.9 ± 0.6	2.9 ± 0.7	2.8 ± 0.8
Total pulmonary resistance (WU)	7.4 ± 3.5	7.6 ± 4.4	7.0 ± 4.2
Pulmonary vascular resistance (WU)	5.0 ± 2.8	5.3 ± 3.4	4.5 ± 3.1
Systemic vascular resistance (WU)	17.5 ± 5.1	18.4 ± 6.1	19.6 ± 6.4
**Blood gases, arterial**			
Hemoglobin (g/dl)	14.0 ± 1.8	14.1 ± 1.6	14.1 ± 1.7
Oxygen saturation (%)	92 ± 3	87 ± 6	98 ± 1
pH	7.45 ± 0.03	7.49 ± 0.05	7.46 ± 0.05
Partial pressure of oxygen (kPa)	9.4 ± 1.8	7.0 ± 1.2	60.5 ± 12.9
Partial pressure of carbon dioxide (kPa)	4.5 ± 0.5	4.2 ± 0.8	4.4 ± 0.8
Lactate (mmol/l)	1.1 ± 0.6	1.0 ± 0.6	0.9 ± 0.5
**Blood gases, mixed venous**			
Oxygen saturation (%)	65 ± 7	62 ± 8	78 ± 6
Partial pressure of oxygen (kPa)	4.6 ± 0.6	4.3 ± 0.5	6.0 ± 0.9
Partial pressure of carbon dioxide (kPa)	5.2 ± 0.6	4.8 ± 0.7	5.2 ± 0.8
**Oxygen content and delivery**			
Oxygen delivery (ml/min)	941 ± 274	890 ± 275	1,024 ± 313
Pulmonary capillary content of oxygen (ml/dl)	–	–	20.8 ± 2.2
Arterial content of oxygen (ml/dl)	17.6 ± 2.3	16.5 ± 2.2	19.9 ± 2.3
Mixed venous content of oxygen (ml/dl)	12.3 ± 2.1	11.8 ± 2.1	14.8 ± 2.1
Shunt fraction (%)	–	–	14.1 ± 5.7
Shunt fraction >5%	–	–	138 (99%)
Shunt fraction >10%	–	–	105 (75%)

*FiO_2_, fraction of inspired oxygen; RHC, right heart catheterization*.

During acute exposure to hyperoxia, univariable mixed models showed a significant reduction in mPAP of 4.4 mmHg and PVR decreased by 0.43 WU. PAWP (−0.5 mmHg) and CI (−0.15 l/min/m^2^) significantly decreased compared with normoxia. PaO_2_ and PmvO_2_ rose (Tables 2, 3). These changes in hemodynamic parameters persisted after adjustment for age, sex, PH-classification, K_CO_, and Qs/Qt in multivariable mixed models (Table 3). The magnitude of these hemodynamic changes remained largely similar if the two large subgroups, PAH and CTEPH, were analyzed separately (Supplementary Tables 1–4).

**Table 3:** Mixed linear regression analysis of hemodynamic parameters dependent on FiO_2_.

**Dependent variable**	**Factor**	**Mean change**	**95% Confidence interval**		***p*-value**
**Univariable analysis:**				
Mean pulmonary artery pressure, mmHg	Hypoxia	0.585	−0.503	1.673	0.292
	Hyperoxia	−4.421	−5.509	−3.334	<0.001*
Pulmonary vascular resistance, WU	Hypoxia	0.353	0.053	0.653	0.021*
	Hyperoxia	−0.427	−0.727	−0.127	0.006*
Cardiac index, l/min/m^2^	Hypoxia	−0.010	−0.109	0.088	0.841
	Hyperoxia	−0.145	−0.243	−0.046	0.004*
Pulmonary artery wedge pressure, mmHg	Hypoxia	−0.541	−0.920	−0.162	0.005*
	Hyperoxia	−0.547	−0.926	−0.168	0.005*
**Multivariable analysis:**					
Mean pulmonary artery pressure, mmHg	Hypoxia	0.246	−0.861	1.353	0.663
	Hyperoxia	−4.452	−5.560	−3.345	<0.001*
	Age	−0.108	−0.242	0.026	0.114
	Female	−0.432	−5.098	4.235	0.856
	CTEPH vs. PAH	2.314	−2.693	7.321	0.365
	Qs/Qt	−0.148	−0.364	0.068	0.179
	K_CO_	−0.137	−0.264	−0.010	0.035*
Pulmonary vascular resistance, WU	Hypoxia	0.351	0.015	0.686	0.041*
	Hyperoxia	−0.398	−0.734	−0.063	0.020*
	Age	−0.013	−0.046	0.019	0.419
	Female	0.629	−0.499	1.756	0.275
	CTEPH vs. PAH	1.193	−0.019	2.404	0.053
	Qs/Qt	−0.025	−0.086	0.036	0.419
	K_CO_	−0.034	−0.064	−0.003	0.034*
Cardiac index, l/min/m^2^	Hypoxia	−0.023	−0.142	0.096	0.705
	Hyperoxia	−0.156	−0.275	−0.037	0.011*
	Age	−0.010	−0.017	−0.003	0.003*
	Female	−0.030	−0.259	0.200	0.799
	CTEPH vs. PAH	−0.359	−0.606	−0.111	0.005*
	Qs/Qt	0.018	0.002	0.334	0.031*
	K_CO_	0.001	−0.006	0.007	0.828
Pulmonary artery wedge pressure, mmHg	Hypoxia	−0.419	−0.823	−0.016	0.042*
	Hyperoxia	−0.538	−0.942	−0.135	0.009*
	Age	0.027	−0.014	0.068	0.192
	Female	0.197	−1.233	1.629	0.787
	CTEPH vs. PAH	−0.093	−1.630	1.444	0.906
	Qs/Qt	−0.022	−0.096	0.052	0.554
	K_CO_	0.013	−0.026	0.052	0.519

Multivariable mixed regression models displaying the mean change (coefficient) with 95% CI of resting hemodynamic parameters dependent on different FiO_2_ values, where hypoxia = FiO_2_ 0.15 and hyperoxia = FiO_2_ 1.0, and adjusted for multiple additional factors. CTEPH, chronic thromboembolic pulmonary hypertension; K_CO_, carbon monoxide transfer coefficient; PAH, pulmonary arterial hypertension; Qs/Qt, right-to-left shunt fraction; statistically significant values are denoted with ^*^.

Univariable regression models adjusted for arterial and mixed venous oxygen partial pressures (independent of administered FiO_2_), revealed that mPAP and PVR were negatively associated with PaO_2_ and PmvO_2_ (Table 4), also when PAH and CTEPH were analyzed separately. Whereas, *CI* was negatively associated with PaO_2_ but not PmvO_2_, PAWP was independent of both (Table 4). Combined in a multivariable mixed linear regression analysis adjusted for age, sex, PH-class, and Qs/Qt, neither mPAP and PVR were significantly inversely associated with PaO_2_, while both were independently associated with PmvO_2_ (Table 4).

**Table 4:** Mixed linear regression analysis of hemodynamic parameters dependent on arterial and mixed oxygen partial pressures, independent of FiO_2_.

**Dependent variable**	**Factor**	**Mean change**	**95% confidence interval**		***p*-value**
**Univariable analysis:**				
Mean pulmonary artery pressure, mmHg	PaO_2_	−0.083	−0.100	−0.065	<0.001*
Pulmonary vascular resistance, WU		−0.011	−0.016	−0.006	<0.001*
Cardiac Index, l/min/m^2^		−0.003	−0.005	−0.001	0.001*
Pulmonary artery wedge pressure, mmHg		−0.002	−0.008	0.004	0.494
Mean pulmonary artery pressure, mmHg	PmvO_2_	−2.668	−3.187	−2.149	<0.001*
Pulmonary vascular resistance, WU		−0.474	−0.620	−0.328	<0.001*
Cardiac Index, l/min/m^2^		−0.021	−0.070	0.029	0.411
Pulmonary artery wedge pressure, mmHg		−0.024	−0.205	0.158	0.799
**Multivariable analysis:**					
Mean pulmonary artery pressure, mmHg	PaO_2_	−0.020	−0.053	0.014	0.257
	PmvO_2_	−2.400	−3.451	−1.348	<0.001*
	Age	−0.132	−0.260	−0.003	0.044*
	Female	−0.211	−4.682	4.260	0.926
	CTEPH vs. PAH	1.392	−3.417	6.201	0.571
	Qs/Qt	−0.108	−0.323	0.106	0.323
	K_CO_	−0.113	−0.236	0.010	0.071
Pulmonary vascular resistance, WU	PaO_2_	0.008	−0.002	0.018	0.097
	PmvO_2_	−0.703	−1.011	−0.395	<0.001*
	Age	−0.020	−0.051	0.010	0.193
	Female	0.732	−0.333	1.796	0.178
	CTEPH vs. PAH	1.047	−0.100	2.194	0.074
	Qs/Qt	−0.007	−0.066	0.052	0.827
	K_CO_	−0.029	−0.059	0.000	0.052
Cardiac index, l/min/m^2^	PaO_2_	−0.007	−0.011	−0.004	<0.001*
	PmvO_2_	0.154	0.049	0.259	0.004*
	Age	−0.009	−0.015	−0.002	0.007*
	Female	−0.048	−0.268	0.172	0.670
	CTEPH vs. PAH	−0.340	−0.578	−0.103	0.005*
	Qs/Qt	0.010	−0.006	0.026	0.224
	K_CO_	0.000	−0.006	0.006	0.988
Pulmonary artery wedge pressure, mmHg	PaO_2_	−0.008	−0.021	0.004	0.590
	PmvO_2_	0.189	−0.192	0.571	0.330
	Age	0.029	−0.012	0.071	0.170
	Female	0.121	−1.322	1.565	0.870
	CTEPH vs. PAH	−0.089	−1.642	1.465	0.911
	Qs/Qt	−0.030	−0.105	0.045	0.435
	K_CO_	0.011	−0.028	0.051	0.590

Multivariable mixed regression models displaying the mean change (coefficient) with 95% CI of resting hemodynamic variables dependent on multiple factors. This model is adjusted for the arterial partial pressure of oxygen (PaO_2_) independent of FiO_2_, due to its strong collinearity with FiO_2_ (variance inflation factor >10). CTEPH, chronic thromboembolic pulmonary hypertension; K_CO_, carbon monoxide transfer coefficient; PAH, pulmonary arterial hypertension; Qs/Qt, right-to-left shunt fraction; statistically significant values (p < 0.05) are denoted with ^*^.

Fraction of right-to-left shunt (Qs/Qt) calculated under hyperoxia was 14.1 ± 5.7%, with 138/139 (99%) subjects showing Qs/Qt levels above 5% and 105/139 (75%) above 10%. There was no significant difference in Qs/Qt between patients with PAH (14.5 ± 5.5%) and CTEPH (14.2 ± 6.7%). The Qs/Qt values could not be calculated in 10 subjects due to missing blood gas values (Table 2). Both mPAP and PVR showed a significant negative association with K_CO_, but were unaffected by age, sex, PH class, and Qs/Qt, while mPAP and PAWP were independent of all adjusting variables, including K_CO_. *CI* showed a positive association with Qs/Qt, a negative association with age and was decreased in patients with CTEPH compared with PAH, but independent of sex (Table 4).

## Discussion

In this large cohort of patients with PAH/CTEPH assessed by RHC, we found that breathing pure oxygen for 10 min significantly reduced mPAP on average by 4.4 mmHg, a reduced size that is remarkable and within the range of vasodilator drugs, along with a decrease in PVR by 0.43 WU, and the size of these hemodynamic changes was similar in the PAH and CTEPH subgroups (27, 28). This finding indicates an acute vasodilatory effect of oxygen and encourages to further explore the therapeutic value of oxygen in pulmonary vascular diseases in short- and long-term settings and may provide an underlying physiological explanation for the benefit from nocturnal or domiciliary oxygen therapy in terms of improvement of exercise capacity and symptoms in PH. Contrarily, the mPAP was unchanged in response to hypoxia, which may indicate that the altered pulmonary vasculature in PH may not reveal HPV, but still dilates to hyperoxia. In regard to the unchanged mPAP and *CI*, the increased PVR with hypoxia was solely driven by a minor but significant decrease in PAWP. The changes of mPAP and PVR under different FiO_2_ were not associated with the size of the Qs/Qt.

### Pulmonary Hemodynamics Under Different FiO_2_

This hitherto largest study investigating pulmonary hemodynamics by RHC in patients with pulmonary vascular diseases showed that changes in FiO_2_ had distinct effects on resting hemodynamics. Under hypoxia, we confirmed our previous and potentially unforeseen finding that in this large collective of patients with PH, hypoxia had no effect on mPAP (6). We did observe an increase in PVR, but as *CI* did not change under hypoxia, the increase in PVR under hypoxia was driven by a minor decrease in PAWP, so that the total pulmonary resistance remained unchanged. However, as PAWP was independent of both PaO_2_ and PmvO_2_, it seems that the change of PAWP was unrelated to the changes in oxygen partial pressures. Moreover, we observed a slight, but significant decrease of PAWP during hyperoxia, further indicating that the changes in PAWP are likely unrelated to FiO_2_ or oxygen partial pressures. We hypothesize that breathing through a tube connected to the airtight mouthpiece might have slightly modified intrathoracic pressures which could have contributed to the slightly lowered venous return and thus the PAWP during both hypoxia and hyperoxia exposure. Thus, the present results indicate that patients with PH with a chronically diseased pulmonary vasculature, acute HPV as found in healthy subjects may be blunted (29–32). Whether this is due to the altered mitochondrial redox signaling or altered response of potassium or calcium channels of diseased pulmonary artery smooth muscle cells in PH remains to be determined (33). It equally remains to be seen, whether long-term exposure to hypoxia, as during mountain travel, would worsen the pulmonary hemodynamics in patients with PH.

On the other hand, when patients with PH breathed pure oxygen (hyperoxia), we observed a large decrease in mPAP and PVR. The decreased PVR is especially remarkable as also the *CI* and the PAWP decreased during oxygen breathing, which would both, in turn, increase the PVR. Both the decrease in CI and PAWP is consistent with previous reports (7). A possible explanation for the largely improved pulmonary hemodynamics with oxygen might be the high FiO_2_ used, both in the present and in previous studies (7). However, a previous study by Leuchte et al. found that oxygen given at a flow rate of 5 l/min to achieve a SpO_2_ > 90% improved mPAP and PVR in patients with PH, suggesting that lower flow rates could already achieve clinically important improvements (8). It is further remarkable, that pure oxygen breathing in this acute setting gave for 10' comparably improved hemodynamics in PAH and CTEPH. Whether oxygen therapy would reveal a beneficial effect on pulmonary hemodynamics and especially functional parameters in long-term studies and which level of FiO_2_ would be sufficient remains to be determined in future well-designed randomized-controlled trials.

The mechanism of action of oxygen therapy in patients with pulmonary vascular disease is not exactly known. A recently published review suggested that acute oxygen induced pulmonary vasodilation is predominantly mediated by alveolar mechanisms and may not even require correction of hypoxemia, although data on mixed venous oxygen content levels were not reported (7). To further explore this mechanism, we calculated linear regression models focusing on the effects of PaO_2_ and PmvO_2_ on pulmonary hemodynamics. We found that mPAP and PVR were inversely and linearly dependent on PaO_2_ and PmvO_2_, with the effect of PmvO_2_ on both mPAP and PVR being greater in magnitude than PaO_2_. While there is no evidence that HPV is both dependent on PaO_2_ and PmvO_2_, with PaO_2_ as directly related to the alveolar PO_2_ being the more important factor (34), these models suggest that the mixed venous hypoxemia plays a role in pulmonary vasoconstriction. Patients with PH reveal mostly normal lung functions so that alveolar hypoxia inducing HPV should not majorly contribute to hypoxemia, which is usually attributed to a low mixed venous oxygenation induced by a low cardiac output (35). In precapillary PH, the culprit vascular lesions that contribute to the increased pressure and resistance are mainly located upstream of the pulmonary capillaries and alveoli before the most gas exchange takes place, although capillary and pulmonary venular remodeling is also found (36, 37). Whether changes in pulmonary precapillary vasculature modify mixed venous oxygen sensing and thus pulmonary vasoconstriction and dilation remain to be investigated.

To summarize, although the duration of exposure to oxygen was short and the FiO_2_ high, our findings may support the therapeutic value of oxygen in pulmonary vascular diseases in a short-term setting and may provide some physiological explanation of the effect of nocturnal or domiciliary oxygen therapy on improvement of exercise capacity and symptoms as previously shown, but future studies are needed to investigate the long-term effects on pulmonary hemodynamics (9, 10).

### The Influence of Diffusion Capacity for Carbon Dioxide (K_CO_) on Pulmonary Hemodynamics

In the multivariable regression analyses, we saw that both mPAP and PVR are negatively associated with the carbon monoxide transfer coefficient (Kco). These findings indicate that patients with more severely compromised pulmonary hemodynamics reveal higher impairments of pulmonary gas exchange, or vice versa, which might be associated with more severe vascular remodeling. Strikingly, even after adjustment for K_CO_, the effect size of both hypoxia and hyperoxia remained the same, indicating that the effect of different FiO_2_ in our regression model remains robust.

### Hemodynamics Dependent on Qs/Qt

Almost all patients (99%) showed Qs/Qt above the physiological threshold of 5 and 75% above the clinically more frequently used threshold of 10% (11). This is comparable with a previous study in which all 87 patients with PH revealed increased Qs/Qt (17). Interestingly, in the present study, changes in resting hemodynamic parameters under hypoxia and hyperoxia were all independent of the size of Qs/Qt. Thus, our data did not support the hypothesis that patients with an increased right-to-left shunt fraction, e.g., through a PFO or IPAVA, would reveal exaggerated responses in mPAP and PVR to hypoxia or hyperoxia, for instance by acting as an escape valve mechanism. This is in line with retrospective studies in subjects with PH and PFO that did not show any association with mPAP or PVR (16–18). However, due to the retrospective nature of the present study, we cannot provide data on the PFO as we do not systematically screen patients with saline contrast echocardiography. As we only calculated Qs/Qt under hyperoxia, we cannot exclude the possibility that Qs/Qt might change depending on the current FiO_2_. Future approaches could include contrast echography under normoxia and hypoxia to detect potential changes in Qs/Qt at different FiO_2_ (38) or investigate whether therapies for pulmonary vascular disease would influence shunt fractions.

### Influence of Age and Sex on Pulmonary Hemodynamics

Adjustment for age, sex, and PH disease classification (PAH vs. CTEPH) (2) in the multivariable regression model revealed that mPAP, PVR, and PAWP were independent of age and PH-classification. *CI* showed a negative association with age.

### Limitations

Exposure to hypoxia and hyperoxia was only of short duration (at least 10 min) and further studies are required to investigate whether longer exposure would consistently change hemodynamics and whether different oxygen fractions used would result in hemodynamic or clinical improvements on long-term. We assumed that a washout period of at least 10 min between different FiO_2_ levels would suffice to avoid a significant carry-over effect of the interventions. Additionally, as all patients received the same order of FiO_2_ intervention (first hypoxia and then hyperoxia), we cannot exclude any order or time effects, which could potentially affect results. For physiological reasons, the Qs/Qt from blood gases can only be calculated under FiO_2_ 1.0 and we do not know whether a herewith-calculated shunt fraction remains stable under different FiO_2_, an assumption that has been questioned (11, 19).

## Conclusion

In this large cohort of patients with PH, acute exposure to hypoxia did not significantly alter mPAP or total pulmonary resistance, which indicates that HPV is blunted in patients with preexisting pulmonary vascular disease. Short-term pure oxygen breathing markedly reduced both mPAP and PVR and supports further exploration into the effect of long-term oxygen treatment on prognosis in pulmonary vascular disease. A large proportion of patients with PH reveal an increased Qs/Qt, which did not affect pulmonary hemodynamics under different FiO_2_.

## Data Availability Statement

The raw data supporting the conclusions of this article will be made available by the authors, without undue reservation.

## Ethics Statement

The studies involving human participants were reviewed and approved by Cantonal Ethics Committee of Zurich (No. 2020–01541). The patients/participants provided their written informed consent to participate in this study.

## Author Contributions

SU is the guarantor and takes responsibility for the content of the manuscript, including the data and analysis. AC contributed to acquiring, analyzing, and interpreting the data, writing and revising the article critically for important intellectual content, and providing final approval of the version to be published. ML, CB, SS, ES, SRS, MF, and KB contributed to data collection and analysis and revising the article critically for important intellectual content. ML and SU conceived the project, contributed to data collection, analysis, interpretation, writing the manuscript, revising the article critically for important intellectual content, and providing final approval of the version to be published. All authors took responsibility for all aspects of the reliability and freedom from bias of the data presented and their discussed interpretation. All authors contributed to the article and approved the submitted version.

## Conflict of Interest

SU reports grants from Johnson and Johnson SA, Switzerland, during the conduct of the study; grants from the Swiss National Science Foundation, grants from Zurich Lung, grants from Orpha Swiss, personal fees from Actelion SA, Switzerland, personal fees from MSD Switzerland, outside the submitted work. The remaining authors declare that the research was conducted in the absence of any commercial or financial relationships that could be construed as a potential conflict of interest.

## Publisher's Note

All claims expressed in this article are solely those of the authors and do not necessarily represent those of their affiliated organizations, or those of the publisher, the editors and the reviewers. Any product that may be evaluated in this article, or claim that may be made by its manufacturer, is not guaranteed or endorsed by the publisher.

## Abbreviations

BMI: body mass index
BP: blood pressure
BSA: body surface area
CI: cardiac Index
CO: cardiac Output
DLCO: diffusing capacity of lung carbon monoxide
CTEPH: chronic thromboembolic pulmonary hypertension
FEV_1_: forced expiration in the first second of expiration
FVC: forced vital capacity
FiO_2_: fraction of inspired oxygen
Hb: hemoglobin
HR: heart rate
IPAVA: intrapulmonary arteriovenous anastomosis
K_CO_: carbon monoxide transfer coefficient
mPAP: mean pulmonary artery pressure
PaCO_2_: arterial partial pressure of carbon dioxide
PAH: pulmonary arterial hypertension
PAO_2_: alveolar partial pressure of oxygen
PaO_2_: arterial partial pressure of oxygen
PAWP: pulmonary artery wedge pressure
P_B_: barometric pressure at the elevation of 490 m
P_H2O_: partial pressure of water at body temperature
PmvO_2_: mixed venous partial pressure of oxygen
PVR: pulmonary vascular resistance
Qs/Qt: fraction of right-to-left shunt
R: respiratory quotient
SaO_2_: arterial saturation of oxygen
ScapO_2_: pulmonary capillary oxygen saturation
SD: standard deviation
SmvO_2_: mixed venous saturation of oxygen
WHO: World Health Organization
WU: wood unit.
