# Supplementary material for: The Impact of Breathing Hypoxic Gas and Oxygen on Pulmonary Hemodynamics in Patients With Pulmonary Hypertension
Source: Front Med (Lausanne). 2022 Feb 11;9:791423. doi: 10.3389/fmed.2022.791423 (PMC8878983; doi:10.3389/fmed.2022.791423)
Supplement: Supplementary file 1 [file Data_Sheet_1.PDF]

Supplementary Table 1: Patient baseline characteristics

	PAH	CTEPH
Number	79	70
Female, n (%)	55 (70)	33 (47)
Age, years	58 (17)	62 (15)
Height, cm	167 (11)	172 (9)
Weight, kg	74 (17)	80 (17)
BMI, kg/m^2	26 (5)	27 (5)
BSA, m^2	1.8 (0.2)	1.9 (0.2)
Pulmonary hypertension classification (WHO)		
1. Pulmonary arterial hypertension	79 (53)	-
1.1. Idiopathic	31 (21)	-
1.2. Hereditable	1 (1)	-
1.4.1. Connective tissue disease	35 (23)	-
1.4.2. HIV Infection	3 (2)	-
1.4.3. Portal hypertension	3 (2)	-
1.4.4. Congenital heart disease	5 (3)	-
1.4.5. Schistosomiasis	1 (1)	-
Incident patients newly diagnosed not yet on therapy:	71 (90)	61 (87)
Pulmonary function tests, % predicted		
FEV_1	82 (20)	86 (17)
FVC	84 (19)	89 (16)
VC	94 (20)	95 (19)
TLC	93 (23)	99 (23)
DLCO	53 (20)	73 (16)

Kco	64 (20)	82 (15)

Data are displayed as mean ± standard deviation or as number (n) with percentage (%). CTEPH: Chronic thromboembolic pulmonary hypertension; DLCO: Diffusion capacity of carbon monoxide; FEV_1: Forced expiration in the first second of expiration; FVC: Forced vital capacity; K_{CO}: Carbon monoxide transfer coefficient; PAH: Pulmonary arterial hypertension; TLC: Total lung capacity; VC: Vital capacity; WHO: World health organization; BMI: Body mass index; BSA: Body surface area.

Supplementary Table 2: Resting hemodynamics and blood gases under normoxic, hypoxic and hyperoxic air breathing (normoxia, hypoxia, hyperoxia)

	**Normoxia (FiO_2 0.21)**		**Hypoxia (FiO_2 = 0.15)**		**Hyperoxia (FiO_2 = 1.0)**	
**Resting hemodynamics during RHC**	**PAH**	**CTEPH**	**PAH**	**CTEPH**	**PAH**	**CTEPH**
Oxygen saturation (%)	94 ± 3	94 ± 4	89 ± 6	90 ± 5	100 ± 1	100 ± 3
Heart rate (min^{-1})	75 ± 12	72 ± 12	78 ± 13	74 ± 14	70 ± 13	67 ± 14
Blood pressure, systolic (mmHg)	130 ± 18	130 ± 15	132 ± 22	131 ± 14	133 ± 21	133 ± 16
Blood pressure, mean (mmHg)	98 ± 13	96 ± 13	100 ± 16	98 ± 13	101 ± 15	99 ± 13
Blood pressure, diastolic (mmHg)	77 ± 12	76 ± 11	79 ±13	78 ± 11	78 ± 12	79 ± 18
Pulmonary artery pressure, systolic (mmHg)	52 ± 20	54 ± 18	53 ± 21	56 ± 21	46 ± 18	48 ± 18
Pulmonary artery pressure, mean (mmHg)	36 ± 13	35 ± 11	37 ± 14	35 ± 12	32 ± 11	31 ± 11
Pulmonary artery pressure, diastolic (mmHg)	24 ± 10	24 ± 8	24 ± 11	22 ± 8	21 ± 9	20 ± 8
Pulmonary artery wedge pressure (mmHg)	12 ± 3	11 ± 3	11 ± 4	11 ± 4	11 ± 4	11 ± 4
Right atrial pressure (mmHg)	9 ± 4	8 ± 4	8 ± 4	7 ± 5	7 ± 4	7 ± 4
Cardiac output (l/min)	5.6 ± 1.4	5.2 ± 1.4	5.7 ± 1.6	5.2 ± 1.4	5.4 ± 1.7	4.9 ± 1.3
Cardiac index ($l/min/m^2$)	3.1 ± 0.7	2.7 ± 0.6	3.1 ± 0.8	2.7 ± 0.6	3.0 ± 0.9	2.5 ± 0.6
Total pulmonary resistance (WU)	6.8 ± 2.9	7.6 ± 4.0	7.1 ± 4.1	7.7 ± 4.7	6.4 ± 3.8	7.2 ± 4.6
Pulmonary vascular resistance (WU)	4.6 ± 2.5	5.1 ± 3.0	5.0 ± 3.4	5.3 ± 3.4	4.2 ± 3.0	4.6 ± 3.2
Systemic vascular resistance (WU)	16.8 ± 14.9	18.1 ± 5.4	17.8 ± 6.5	18.9 ± 5.6	18.5 ± 5.8	20.4 ± 6.8
**Blood gases, arterial**						
Hemoglobin (g/dl)	13.7 ± 1.7	14.3 ± 1.8	13.7 ± 1.6	14.5 ± 1.4	13.7 ± 1.8	14.4 ± 1.3
Oxygen saturation (%)	93.0 ± 3.4	91.9 ± 3.5	87.2 ± 5.9	86.6 ± 5.2	98.3 ± 1.3	98.5 ± 1.4
pH	7.4 ± 0.0	7.4 ± 0.0	7.5 ± 0.0	7.5 ± 0.0	7.5 ± 0.1	7.5 ± 0.1
Partial pressure of oxygen (kPa)	9.8 ± 1.9	9.0 ± 1.6	7.1 ± 1.3	6.8 ± 1.1	61.8 ± 12.8	59.6 ± 13.2
Partial pressure of carbon dioxide (kPa)	4.5 ± 0.6	4.5 ± 0.5	4.2 ± 0.7	4.2 ± 0.8	4.5 ± 0.8	4.3 ± 0.7

Lactate (mmol/l)	1.2 ± 0.7	1.1 ± 0.5	1.0 ± 0.8	0.9 ± 0.3	0.9 ± 0.6	0.9 ± 0.3
**Blood gases, mixed venous**						
Oxygen saturation (%)	65.4 ± 7.5	64.8 ± 7.0	63.1 ± 9.0	61.2 ± 7.7	79.0 ± 6.0	77.2 ± 6.1
Partial pressure of oxygen (kPa)	4.8 ± 0.7	4.5 ± 0.4	4.4 ± 0.6	4.2 ± 0.4	6.2 ± 0.9	5.8 ± 1.0
Partial pressure of carbon dioxide (kPa)	5.3 ± 0.6	5.2 ± 0.6	4.9 ± 0.7	4.7 ± 0.7	5.3 ± 0.9	5.1 ± 0.8
**Oxygen content and delivery**						
Oxygen delivery (ml/min)	968 ± 300	921 ± 249	924 ± 303	875 ± 253	1071 ± 354	1000 ± 271
Pulmonary capillary content of oxygen (ml/dl)	-	-	-	-	20.3 ± 2.4	21.3 ± 1.8
Arterial content of oxygen (ml/dl)	17.3 ± 2.2	17.9 ± 2.4	16.1 ± 2.2	17.0 ± 2.1	19.5 ± 2.4	20.4 ± 2.0
Mixed venous content of oxygen (ml/dl)	12.2 ± 2.1	12.5 ± 2.1	11.7 ± 2.1	12.0 ± 2.1	14.7 ± 2.2	15.1 ± 2.0
Shunt fraction (%)	-	-	-	-	14.5 ± 5.4	14.1 ± 6.7
Shunt fraction >5%	-	-	-	-	71 (100%)	67 (99%)
Shunt fraction >10%	-	-	-	-	55 (77%)	50 (74%)

CTEPH: Chronic thromboembolic pulmonary hypertension; FiO_2: Fraction of inspired oxygen; RHC: right heart catheterization; PAH: Pulmonary arterial hypertension.

Supplementary table 3: Mixed linear regression analysis of hemodynamic parameters dependent on FiO_2

**Univariable analysis:**		**Pulmonary arterial hypertension**				**Chronic thromboembolic pulmonary hypertension**			
Dependent variable	Factor	Mean change	95% Confidence interval		p value	Mean change	95% Confidence interval		p value
Mean pulmonary artery pressure, mmHg	Hypoxia	0.695	-0.703	2.093	0.330	0.468	-1.221	2.156	0.587
	Hyperoxia	-4.268	-5.667	-2.870	<0.001*	-4.584	-6.273	-2.896	<0.001*
Pulmonary vascular resistance, WU	Hypoxia	0.402	-0.033	0.838	0.070	0.300	-0.114	0.714	0.155
	Hyperoxia	-0.392	-0.827	0.044	0.078	-0.465	-0.879	-0.051	0.028*
Cardiac index, $l/min/m^2$	Hypoxia	0.022	-0.145	0.189	0.796	-0.044	-0.145	0.057	0.392
	Hyperoxia	-0.083	-0.250	0.084	0.329	-0.210	-0.311	-0.109	<0.001*
Pulmonary artery wedge pressure, mmHg	Hypoxia	-0.588	-1.076	-0.010	0.018*	-0.421	-1.016	0.175	0.166
	Hyperoxia	-0.625	-1.113	-0.137	0.012*	-0.395	-0.990	0.201	0.194
**Multivariable analysis**		**Pulmonary arterial hypertension**				**Chronic thromboembolic pulmonary hypertension**			
Dependent variable	Factor	Mean change	95% Confidence interval		p value	Mean change	95% Confidence interval		p value
Mean pulmonary artery pressure, mmHg	Hypoxia	0.132	-1.473	1.738	0.872	0.379	-1.142	1.901	0.625
	Hyperoxia	-4.471	-6.076	-2.865	<0.001*	-4.431	-5.952	-2.910	<0.001*
	Age	-0.211	-0.386	-0.036	0.018*	0.107	-0.118	0.332	0.352
	Female	-2.254	-8.608	4.099	0.487	4.733	-2.732	12.198	0.214
	Qs/Qt	-0.048	-0.567	0.471	0.857	-0.167	-0.392	0.058	0.145
	K_{CO}	-0.152	-0.304	-0.001	0.049*	-0.042	-0.288	0.204	0.737
Pulmonary vascular resistance, WU	Hypoxia	0.431	-0.077	0.938	0.096	0.257	-0.170	0.684	0.238
	Hyperoxia	-0.324	-0.831	0.184	0.212	-0.486	-0.913	-0.060	0.026*
	Age	0.423	-1.025	1.871	0.567	1.630	-0.299	3.560	0.098
	Female	-0.044	-0.084	-0.004	0.030*	0.042	-0.017	0.100	0.161
	Qs/Qt	0.016	-0.110	0.141	0.805	-0.040	-0.102	0.022	0.210
	K_{CO}	-0.040	-0.075	-0.005	0.025*	-0.012	-0.075	0.052	0.718
Cardiac index, $l/min/m^2$	Hypoxia	-0.006	-0.201	0.190	0.953	-0.043	-0.166	0.080	0.492
	Hyperoxia	-0.102	-0.297	0.094	0.309	-0.219	-0.342	-0.096	0.001*

	Age	-0.056	-0.393	0.282	0.747	-0.126	-0.461	0.209	0.461
	Female	-0.010	-0.019	0.000	0.044*	-0.014	-0.024	-0.004	0.007*
	Qs/Qt	0.032	0.001	0.062	0.042*	0.010	-0.005	0.026	0.192
	K_{CO}	0.003	-0.005	0.011	0.445	-0.006	-0.017	0.005	0.264
Pulmonary artery wedge pressure, mmHg	Hypoxia	-0.609	-1.137	-0.081	0.024*	-0.455	-1.080	0.169	0.153
	Hyperoxia	-0.565	-1.093	-0.037	0.036*	-0.248	-0.873	0.376	0.436
	Age	-0.431	-2.237	1.375	0.640	1.482	-1.072	4.036	0.255
	Female	0.034	-0.016	0.084	0.178	0.046	-0.031	0.123	0.242
	Qs/Qt	-0.119	-0.271	0.033	0.124	0.006	-0.082	0.093	0.902
	K_{CO}	0.009	-0.034	0.053	0.673	0.044	-0.040	0.128	0.305

Multivariable mixed regression models displaying the mean change (coefficient) with 95% confidence interval of resting hemodynamic parameters dependent on different FiO_2 values, where hypoxia = FiO_2 0.15 and hyperoxia = FiO_2 1.0, and adjusted for multiple additional factors; K_{CO}: carbon monoxide transfer coefficient; Qs/Qt: right-to-left shunt fraction; Statistically significant values are denoted with *.

Supplementary Table 4: Mixed linear regression analysis of hemodynamic parameters dependent on PaO_2 and $PmvO_2$

**Univariable analysis:**		**Pulmonary arterial hypertension**				**Chronic thromboembolic pulmonary hypertension**			
Dependent variable	Factor	Mean change	95% Confidence interval		p value	Mean change	95% Confidence interval		p value
Mean pulmonary artery pressure, mmHg	PaO_2	-0.083	-0.107	-0.059	<0.001*	-0.082	-0.109	-0.056	<0.001*
Pulmonary vascular resistance, WU		-0.011	-0.018	-0.004	0.003*	-0.011	-0.018	-0.004	0.001*
Cardiac Index, $l/min/m^2$		-0.003	-0.005	-0.001	0.076	-0.003	-0.005	-0.002	<0.001*
Pulmonary artery wedge pressure, mmHg		-0.005	-0.012	-0.003	0.231	0.001	-0.009	0.010	0.947
Mean pulmonary artery pressure, mmHg	$PmvO_2$	-2.854	-3.518	-2.189	<0.001*	-2.462	-3.273	-1.651	<0.001*
Pulmonary vascular resistance, WU		-0.507	-0.710	-0.305	<0.001*	-0.431	-0.642	-0.220	<0.001*
Cardiac Index, $l/min/m^2$		0.003	-0.076	0.082	0.943	-0.056	-0.110	-0.003	0.037*
Pulmonary artery wedge pressure, mmHg		-0.075	-0.303	0.153	0.519	0.037	-0.252	0.326	0.801
**Multivariable analysis**		**Pulmonary arterial hypertension**				**Chronic thromboembolic pulmonary hypertension**			
Dependent variable	Factor	Mean change	95% Confidence interval		p value	Mean change	95% Confidence interval		p value
Mean pulmonary artery pressure, mmHg	PaO_2	-0.006	-0.051	0.040	0.814	-0.042	-0.094	0.010	0.112
	$PmvO_2$	-2.745	-4.089	-1.402	<0.001*	-1.731	-3.483	0.021	0.053
	Age	-2.095	-8.135	3.945	0.497	4.818	-2.413	12.049	0.192
	Female	-0.238	-0.404	-0.072	0.005*	0.086	-0.132	0.305	0.439
	Qs/Qt	0.032	-0.468	0.533	0.900	-0.156	-0.388	0.076	0.187
	K_{CO}	-0.122	-0.267	0.024	0.101	-0.036	-0.274	0.202	0.768
Pulmonary vascular resistance, WU	PaO_2	0.013	-0.001	0.027	0.061	0.002	-0.013	0.016	0.843
	$PmvO_2$	-0.824	-1.228	-0.421	<0.001*	-0.505	-0.992	-0.019	0.042*
	Age	0.563	-0.746	1.871	0.399	1.667	-0.220	3.554	0.083
	Female	-0.052	-0.089	-0.016	0.005*	0.036	-0.021	0.093	0.216
	Qs/Qt	0.040	-0.076	0.155	0.499	-0.029	-0.092	0.035	0.382
	K_{CO}	-0.034	-0.066	-0.002	0.039*	-0.011	-0.073	0.051	0.736
Cardiac index, $l/min/m^2$	PaO_2	-0.009	-0.014	-0.004	0.001*	-0.004	-0.008	0.000	0.069
	$PmvO_2$	0.210	0.061	0.359	0.006*	0.013	-0.122	0.148	0.853

	Age	-0.075	-0.392	0.242	0.643	-0.138	-0.472	0.195	0.417
	Female	-0.008	-0.017	0.001	0.066	-0.014	-0.024	-0.004	0.007*
	Qs/Qt	0.023	-0.006	0.053	0.123	0.006	-0.010	0.022	0.437
	K_{CO}	0.002	-0.006	0.010	0.636	-0.006	-0.017	0.005	0.264
Pulmonary artery wedge pressure, mmHg	PaO_2	-0.007	-0.022	0.008	0.339	-0.012	-0.033	0.009	0.251
	$PmvO_2$	0.094	-0.349	0.537	0.677	0.420	-0.286	1.126	0.243
	Age	-0.562	-2.388	1.264	0.546	1.475	-1.106	4.056	0.263
	Female	0.036	-0.014	0.087	0.160	0.050	-0.029	0.128	0.213
	Qs/Qt	-0.122	-0.276	0.032	0.122	-0.009	-0.101	0.082	0.846
	K_{CO}	0.007	-0.037	0.051	0.746	0.044	-0.041	0.128	0.315

Multivariable mixed regression models displaying the mean change (coefficient) with 95% confidence interval of resting hemodynamic variables dependent on multiple factors. This model is adjusted for the arterial partial pressure of oxygen (PaO2) independent of FiO2, due its strong collinearity with FiO2 (variance inflation factor >10). KCO: carbon monoxide transfer coefficient; Qs/Qt: right-to-left shunt fraction; statistically significant values (p<0.05) are denoted with *.
